# Identifying priority areas to support primary care engagement in breast cancer survivorship care: A Delphi study

**DOI:** 10.1002/cam4.7219

**Published:** 2024-04-30

**Authors:** Lisa Mikesell, Denalee M. O'Malley, Rachel T. Kurtzman, Jenna Howard, Benjamin Bates, Jennifer R. Hemler, Sarah J. Fadem, Jeanne M. Ferrante, Alicja Bator, Shawna V. Hudson, Benjamin F. Crabtree

**Affiliations:** ^1^ School of Communication and Information Rutgers University New Brunswick New Jersey USA; ^2^ Institute for Health, Health Care Policy, and Aging Research Rutgers University New Brunswick New Jersey USA; ^3^ Department of Family Medicine Rutgers Robert Wood Johnson Medical School New Brunswick New Jersey USA; ^4^ Rutgers Cancer Institute of New Jersey New Brunswick New Jersey USA; ^5^ NORC at The University of Chicago Chicago Illinois USA; ^6^ Department of Medicine, Division of General Internal Medicine Rutgers Robert Wood Johnson Medical School New Brunswick New Jersey USA

**Keywords:** breast cancer, communication, Delphi panel, primary care, survivorship care

## Abstract

**Introduction:**

Existing approaches in cancer survivorship care delivery have proven to be insufficient to engage primary care. This study aimed to identify stakeholder‐informed priorities to improve primary care engagement in breast cancer survivorship care.

**Methods:**

Experts in U.S. cancer survivorship care delivery were invited to participate in a 4‐round online Delphi panel to identify and evaluate priorities for defining and fostering primary care's engagement in breast cancer survivorship. Panelists were asked to identify and then assess (ratings of 1–9) the *importance* and *feasibility* of priority items to support primary care engagement in survivorship. Panelists were asked to review the group results and reevaluate the *importance* and *feasibility* of each item, aiming to reach consensus.

**Results:**

Respondent panelists (*n* = 23, response rate 57.5%) identified 31 priority items to support survivorship care. Panelists consistently rated three items most important (scored 9) but with uncertain feasibility (scored 5–6). These items emphasized the need to foster connections and improve communication between primary care and oncology. Panelists reached consensus on four items evaluated as important and feasible: (1) educating patients on survivorship, (2) enabling screening reminders and monitoring alerts in the electronic medical record, (3) identifying patient resources for clinicians to recommend, and (4) distributing accessible reference guides of common breast cancer drugs.

**Conclusion:**

Role clarity and communication between oncology and primary care were rated as most important; however, uncertainty about feasibility remains. These findings indicate that cross‐disciplinary capacity building to address feasibility issues may be needed to make the most important priority items actionable in primary care.

## 1 INTRODUCTION

Breast cancer is the most prevalent cancer among women, affecting over 4 million women in the U.S. and represents the largest population of longer‐term cancer survivors.^1^ While the overall five‐year and 15‐year relative survival rate for breast cancers are 91% and 80%, respectively,^1^ women with a history of breast cancer face considerable medical, physical, and psychosocial late effects that persist beyond treatment.^2^ Longer‐term effects can emerge years later and include cardiac toxicities, which manifest on average 7 years after diagnosis and are associated with higher risk of cardiovascular mortality.^3^, ^4^, ^5^ A recent study across 33 states from 2016 to 2020 reported that primary care was involved in care delivery for 65.4% of breast cancer survivors.^6^ Primary care clinicians are also willing to participate in survivorship care.^6^ Moreover, evidence suggests that primary care‐engaged survivorship care models are cost‐effective and as efficacious in identifying recurrence as oncology‐led models.^7^ However, their adoption continues to flounder in the U.S. Identified barriers to primary care delivery of survivorship care include uncertainty about roles, lack of training, and inaccessibility of documentation detailing the needs of patients with a history of cancer.^8^

The idea that primary care needs to be engaged is not new. It was a central focus of the Institute of Medicine's (now the National Academy of Medicine) seminal survivorship report 17 years ago, outlining recommendations that described the need to engage primary care and strategies to support primary care in survivorship care.^9^, ^10^ In 2016, the American Cancer Society and American Society of Clinical Oncology published recommendations to guide primary care in the follow‐up of longer‐term cancer survivors.^11^ In the U.S., the engagement of primary care in survivorship delivery may be hampered by the weak and under‐resourced primary care system infrastructure.^9^, ^12^ The systemic shortcomings of U.S. primary care have warranted the publication of two separate National Academy of Medicine reports (1996 and 2021), the latter of the two acknowledging that little to no progress has emerged since the first.^12^ After the seminal survivorship report, survivorship care plans to inform primary care and clinical guidelines outlining care needs were the major national strategies to support primary care engagement.^13^ Barriers to care plan implementation include lack of resources and systems to support their development and distribution.^13^, ^14^

Despite the evidence supporting primary care engagement in the care for patients with a history of breast cancer, approaches for improving survivorship care have tended to be onco‐centric. As a result, these efforts have done little to translate survivorship care delivery into the realities of U.S. primary care. For example, a comparative case study describing 12 primary care practices with advanced workforces (e.g., sampled from a national registry of workforce innovators) found that none of these clinics had implemented survivorship clinical services. One of the barriers identified from these 12 practices was the lack of actionable information to guide patient care.^15^ Hence, the literature has encouraged participatory research that emphasizes the need to understand the U.S. primary care context, capacity, and receptivity to address the translational lag of primary care engaged survivorship care.^10^, ^16^ Given the onco‐centric approaches identified in the extant literature, this study intentionally centers on the context and priorities of primary care and builds from insights gleaned from expert interviews of Phase I of a larger project focused on addressing the barriers of translating survivorship care into U.S. primary care clinics. The larger project aims to develop an intervention to be implemented and evaluated in a hybrid type 1 effectiveness‐implementation cluster randomized design in 26 primary care practices.^17^ Phase 1 interviews explored breast cancer survivorship care experts' current working models of care and implementation experiences. This study phase convened a Delphi panel of primary care‐engaged survivorship experts to identify primary care‐centered priorities to enhance breast cancer care and reflect on their value according to their collective wisdom. The next phase of this project aims to tailor these priority items identified by primary‐engaged survivorship care experts to clinic contexts in order to more carefully consider strategies for implementation.

## 2 MATERIALS AND METHODS

The Delphi technique is used to explore the existence of consensus among groups of knowledgeable individuals with vested interests in a problem area to develop research or policy agendas and identify values and preferences.^18^, ^19^, ^20^ Drawing on the collective knowledge of experts has been argued to lead to more accurate results than relying on individual judgment.^21^, ^22^ Delphi panels enable panelists to assess the value of available knowledge, whether that knowledge stems from prior scientific findings and/or from the collective expertise of panelists.^22^ The Delphi technique is most frequently employed where uncertainty or incomplete information exists.^22^, ^23^, ^24^ As significant uncertainty persists regarding how to transfer knowledge from cancer care to primary care and because challenges may stem from the onco‐centric efforts to date, consensus‐building approaches leveraging the clinical judgment of a diverse group of primary‐care engaged survivorship experts to evaluate current knowledge related to clinical questions can help establish the priority areas that may best enhance primary care involvement in the care of patients with a history of breast cancer.

### 2.1 Expert panel participants

As is common in Delphi panels,^18^, ^19^ we purposively sampled cancer survivorship experts. We considered individuals who were currently participating in and/or developing primary care engaged cancer survivorship programs in the U.S. to possess expert knowledge about (1) the value of primary care's engagement in survivorship care and (2) strategies required for primary care to participate effectively in the care of patients with a history of breast cancer. We identified expert panelists using three approaches: (1) recommendations from the larger project's scientific advisory committee members and consultants who include experts in survivorship care; (2) snowball sampling, that is, recommendations from individuals we identified as survivorship experts working at the cutting edge of survivorship care and whom we interviewed during Phase I of the study; and (3) internet searches of leading cancer survivorship programs in the U.S. Because Delphi panels with multiple stakeholder groups have been found to generate more accurate results,^19^, ^20^, ^25^ our sampling approach intentionally sought diverse representation among panelists: primary care clinicians; oncology care clinicians; patients, survivors, and patient advocates; researchers (i.e., those testing models of care for survivors); and administrators.

### 2.2 Panel design

**FIGURE 1:**
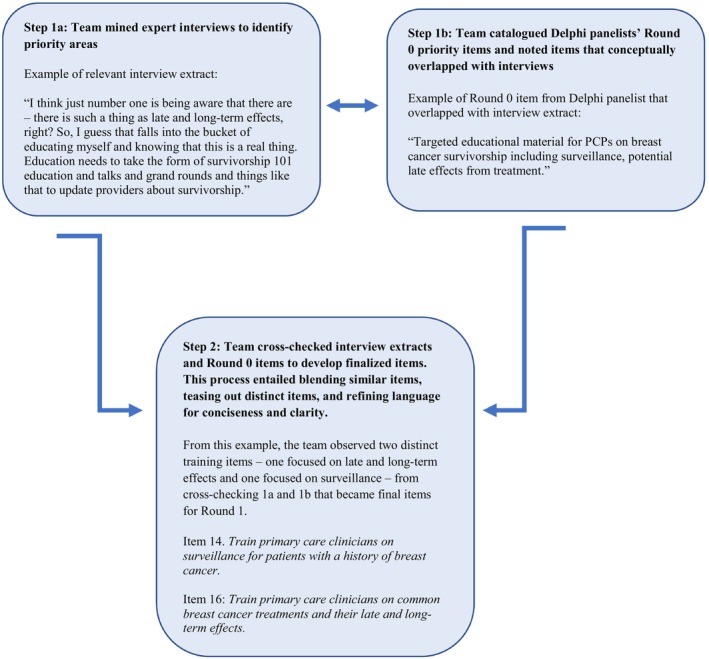
An illustration of generating a Round 1 Delphi item from expert interviews and Round 0 priority recommendations.

This Delphi panel included four rounds. Data was collected between 8/8/2022 and 9/30/2022. In Round 0, we asked panelists to describe priority areas when they were asked to “identify what you think is needed for primary care to deliver quality survivorship care for patients with a history of breast cancer.” We intentionally assessed primary care involvement broadly rather than using a specific survivorship care delivery model as previous research indicates limited adoption of any of the models described in the literature.^15^, ^26^ In Round 0, panelists were able to describe their recommendations without word limits in an open text box format.^27^ From Round 0, 77 recommendations were generated. Additionally, prior to the Delphi panel, an initial list of 46 priority areas was developed from 40 in‐depth interviews^28^ with diverse stakeholders with interest in survivorship (*n* = 32) as well as primary care clinicians (*n* = 8). Each of the 77 panelist‐generated items was cross‐checked against the initial list of 46 items developed from Phase I interviews. To finalize the list of priority items for Round 1, the research team consolidated items if redundant. Priority items that conceptually overlapped or those that included multiple priority items were refined and clarified in the final item description (see Figure 1). This process resulted in 31 unique priority items presented in the subsequent rounds. The research team organized the 31 priority items into six thematic categories: (1) primary care practice training (*n* = 7); (2) resource development and distribution (*n* = 6); (3) oncology‐primary care connections (*n* = 5); technological solutions (*n* = 5); patient education (*n* = 4); and system‐level strategies (*n* = 4). However, priority items were presented to panelists as independent items to not influence their evaluations.

Given the volume of items, the initial goal of Round 1 was to reduce the number of items for the subsequent evaluation rounds. While not a traditional step in the Delphi process, we added this step due to concerns about participant burden and attrition.^29^ In Round 1, we asked panelists to prioritize the “top 10 recommended strategies most crucial for supporting primary care engagement in the care for patients with a history of breast cancer.” Any unranked items from Round 1 were eliminated from the subsequent evaluation rounds. We designed the study to include two evaluation rounds to avoid forced consensus due to participant burnout.^29^, ^30^ In Round 2, we asked panelists to assess each of the items from the Round 1 consolidated list of items on a 9‐point Likert‐type scale along two dimensions: *importance* (how likely the strategy is to improve breast cancer survivorship care and/or adequately resolve a challenge with survivorship care delivery in primary care settings) and *feasibility* (how likely it is the strategy can be implemented in practice). In Round 3, panelists were asked to review the group results (medians, IQRs) and reevaluate the importance and feasibility of each item in an effort to reach consensus. Panelists were paid $50 for each evaluation round (i.e., Rounds 2 and 3), which they completed up to $100. The Rutgers University IRB approved the study protocol (Pro 2021000838), and electronic informed consent was obtained from all participants.

### 2.3 Data analysis

To determine the final group evaluation for each priority item,^31^ we applied the RAND/UCLA Appropriateness Method (RAM) Manual because it provides a validated approach to consensus for nine‐point Likert scales.^32^ A priority item was considered important or feasible if: (a) less than a third of all responses were in the lower tertile of response categories (response options 1–3) and in the upper tertile (response options 7–9) (i.e., responses were not bimodally distributed) and (b) the group median fell into the upper tertile (categories 7–9). If the distribution was not bimodal and the group median fell into the lower tertile (response options 1–3), the group decision was considered not important or not feasible. If the median fell into the middle tertile (response options 4–6), the decision was considered “uncertain.” We analyzed evaluation data for each priority item separately.^32^

## 3 RESULTS

We contacted 40 survivorship experts via email and received 24 (60.0%) replies. One panelist dropped due to unavailability, leaving 23 participants, which is an adequate size for a Delphi panel.^24^, ^33^ All 23 experts participated in Rounds 0 and 1. Participation rates dropped in Round 2 (*n* = 15, 65.2%) and rose in Round 3 (*n* = 20, 87.0%), which exceeds the expected 45%–50% participation rate.^33^, ^34^ Panelists who participated in only Round 3 (*n* = 5) were analyzed separately from those who participated in both rounds. We decided to allow these panelists to participate in Round 3 because they included three of the eight participating oncologists, a perspective we wanted to capture. Participant demographics are described in Table 1.

**TABLE 1:** Participant characteristics (*N* = 23).

			Round 3 Only (*N* = 5)^b^
Age, years (M)	45.7		33
Race^a^	*N* (%)		*N*
Asian	3 (13)		0
Asian American	4 (17.4)		0
White	15 (65.2)		1
Other	2 (8.7)	Indian‐American, Afro‐Caribbean	East‐Indian American
Ethnicity			
Hispanic or Latino/a	3 (13)		0
Not Hispanic or Latino/a	20 (87)		2
Gender			
Male	6 (26.1)		1
Female	17 (73.9)		1
Role^a^		Mean Years in Role	
Primary Care	12 (52.2)	12.7	1
Oncology Care	8 (34.8)	13.5	4
Patient/Patient Advocate/Survivor	1 (4.3)	10	0
Researcher	7 (30.4)	20.9	0
Administration	4 (17.4)	14	0
Health Care Setting^a^			
Primary Care	12 (52.2)		1
Oncology	10 (43.5)		1
Other	1 (4.3)	Contract research agency	0
Not Applicable	1 (4.3)		0
Practice Setting			
Physician owned	2 (8.7)		0
Hospital health system affiliated	9 (39.1)		1
University affiliated	8 (34.8)		1
Publicly sponsored (e.g., FQHC, county, city)	2 (8.7)		0
Not Applicable	2 (8.7)		0

^a^ Respondents allowed to select more than one response.

^b^ Three participants provided no demographic information.

Panelists predominantly identified as White (65.2%), non‐Hispanic (87.0%), and female (73.9%). Primary care clinicians represented the largest professional group assessed (52.2%), averaging 12.7 years in these roles. Similar proportions of oncology clinicians (34.8%) and researchers (30.4%) participated. Administrators (17.4%) and patients/patient advocates/survivors (4.3%) had the smallest representation. Panelists reported working in primary care (52.2%) and oncology (43.5%) health care settings, most frequently in hospital health systems (39.1%) and university‐affiliated practices (34.8%). Round 3 only panelists included three oncologists, one oncology staff, and one primary care clinician.

Panelists reviewed the 31 priority items in Round 1, and after ranking their top ten items, only two items remained unranked and were dropped from subsequent rounds:

- Work with oncology to educate patients about the need to continue to see their primary care physician (strategy #30 in Table 2).
- Implement lunch‐and‐learn sessions with staff and patients/caregivers about cancer survivorship (#31).

**TABLE 2:** Panelist evaluations of importance and feasibility.

	Priority area	Thematic category	Importance (Median, range 1–9)		Feasibility (Median, range 1–9)	
			Round 2 (*n* = 15)	Round 3 (*n* = 15)	Round 2 (*n* = 15)	Round 3 (*n* = 15)
1	Define clear roles and responsibilities for both oncology and primary care for patients with a history of breast cancer	Onc‐PC Connections	9 Important	9 (8.5) Important	*5 Uncertain	6 (5) Uncertain
2	Train oncologists about how primary care can address survivorship care needs and what primary care needs from oncology	Onc‐PC Connections	9 Important	9 (8) Important	*5 Uncertain	5 (5.5) Uncertain
3	Create clear pathways for communicating and sharing information with oncology (how to connect, who to reach out to, when to reach out, what information to share)	Onc‐PC Connections	*9 Important	9 (9) Important	4 Uncertain	5 (6) Uncertain
4	Educate patients on survivorship (risk education, screening/secondary cancers, what symptoms to pay attention to)	Patient Education	8 Important	8 (9) Important	*7 Feasible	7 (7.5) Feasible
5	Enable screening reminders, monitoring alerts, and automated messaging within EHRs for cancer survivors in primary care	Tech Solutions	8 Important	8 (7) Important	*7 Feasible	7 (8) Feasible
6	Identify available resources for primary care clinicians to recommend to patients/families (e.g., behavioral health programs, smoking cessation, fertility preservation clinics)	Resource Development/Distribution	*8 Important	8 (6) Important	*6 Uncertain	7 (6.5) Feasible
7	Standardize what information is included in patients' cancer history in EHR	Tech Solutions	*8 Important	8 (7.5) Important	*6 Uncertain	6.5 (6.5) Uncertain
8	Educate patients on strategies about survivorship care with primary care clinicians and how to communicate their survivorship needs with primary care clinicians	Patient Education	8 Important	8 (8) Important	*6 Uncertain	6 (7.5) Uncertain
9	Work with oncology to develop primary care‐friendly care plans that include treatment summaries and surveillance needed	Onc‐PC Connections	8 Important	8 (6.5) Important	*5 Uncertain	5 (6) Uncertain
10	Train primary care clinicians on breast cancer survivorship guidelines	Primary Care Practice Training	*7 Important	7.5 (8) Important	*4 Uncertain	5 (5.5) Uncertain
11	Develop and/or distribute accessible clinician reference guides detailing common breast cancer drugs and effects	Resource Development/Distribution	7 Important	7 (8) Important	7 Feasible	8 (7) Feasible
12	Add patient‐reported outcomes to patient intake forms that include questions about history of cancer, physical side effects, and psychosocial needs	Resource Development/Distribution	*7 Important	7 (7.5) Important	*5 Uncertain	6 (7) Uncertain
13	Train primary care clinicians on screenings for patients with a history of breast cancer	Primary Care Practice Training	7 Important	7 (8) Important	5 Uncertain	5 (5.5) Uncertain
14	Train primary care clinicians on surveillance for patients with a history of breast cancer	Primary Care Practice Training	*7 Important	7 (8) Important	5 Uncertain	5 (5.5) Uncertain
15	Develop risk‐stratification guidelines for breast cancer survivors based on type/stage of cancer and treatments received	Resource Development/Distribution	7 Important	7 (7.5) Important	5 Uncertain	5.5 (6) Uncertain
16	Train primary care clinicians on common breast cancer treatments and their late and long‐term effects	Primary Care Practice Training	7 Important	7 (7) Important	5 Uncertain	5 (5) Uncertain
17	Work with oncology to engage primary care during acute treatment	Onc‐PC Connections	*7 Important	7 (5.5) Important	*5 Uncertain	5 (5.5) Uncertain
18	Provide managers/coordinators to facilitate survivorship care, including screenings, preparing orders, constructing patient history, outlining care needs	System‐level strategies	*7 Important	7 (7) Important	3 Not feasible	3 (5) Not feasible
19	Design primary care clinician‐friendly templates for follow‐up plans in EHR	Tech Solutions	6 Uncertain	6 (7) Uncertain	*7 Feasible	7 (7) Feasible
20	Train primary care clinicians on appropriate billing strategies for longer survivorship visits	Primary Care Practice Training	*5 Uncertain	6 (8) Uncertain	7 Feasible	6 (6) Uncertain
21	Document consistently where history of cancer is located in EHR (e.g., in problem list, patient history) and which diagnosis codes to use	Tech Solutions	6 Uncertain	6 (6.5) Uncertain	7 Feasible	6 (7) Uncertain
22	Develop/research existing point of care resources for survivorship care (e.g., a smartphone app)	Resource Development/Distribution	*5 Uncertain	*6 (6) Uncertain	7 Feasible	6 (6) Uncertain
23	Develop decision trees (like those used for chest pain) to help primary care clinicians recognize patient symptoms that may stem from cancer treatment	Resource Development/Distribution	*6 Uncertain	6 (5) Uncertain	*6 Uncertain	6 (6) Uncertain
24	Implement a project ECHO on breast cancer survivorship for primary care clinicians	Primary Care Practice Training	7 Important	*6 (7) Uncertain	4 Uncertain	5 (5) Uncertain
25	Identify practice champions and train them to be cancer survivorship experts/dedicated survivorship providers in primary care	System‐level strategies	*7 Important	6 (6) Uncertain	5 Uncertain	5 (6) Uncertain
26	Train primary care clinicians on how to use point of care resources to find information on breast cancer survivorship care	Primary Care Practice Training	6 Uncertain	6 (7) Uncertain	5 Uncertain	5 (5.5) Uncertain
27	Develop a patient registry for cancer survivors in the EHR	Tech Solutions	*3 Not important	5 (8) Uncertain	*6 Uncertain	6 (6) Uncertain
28	Provide primary care clinicians blocked time in schedules dedicated for cancer survivors	System‐level strategies	*5 Uncertain	5 (6) Uncertain	*3.5 Not feasible	*3 (5) Not feasible
29	Implement patient support groups/group visits for breast cancer survivors in primary care	Patient Education	4 Uncertain	*3.5 (4.5) Not important	3 Not feasible	3.5 (4) Not feasible
30	Work with oncology to educate patients about the need to continue to see their PCP	Patient Education	Unranked in Round 1			
31	Implement lunch‐and‐learn sessions with staff and patients/caregivers about cancer survivorship	System‐level strategies	Unranked in Round 1			

*Note*: Numbers in parentheses: Median value, range 1–9 of panelists who participated in Round 3 only (*n* = 5) but did not provide independent assessments in Round 2; they were analyzed separately. * = panelists did not reach consensus based on the RAND/UCLA Appropriateness Method described in the methods section. ECHO = Extension for Community Healthcare Outcomes, which consists of a virtual learning community of primary care physicians, specialists, and staff who would join a series of 60‐min Zoom sessions discussing real‐life patient scenarios.

Abbreviation: PCP, primary care provider.

Table 2 shows panelists' assessments of the importance and feasibility of the remaining 29 items that were evaluated in Rounds 2 and 3.

### 3.1 Consensus

Although panelists did not reach consensus for many items in the initial round of evaluations, by the end of Round 3, consensus was reached for most items. They did not reach consensus on feasibility for one item after completing the final evaluation round: *Provide primary care clinicians blocked time in schedules dedicated for cancer survivors* (strategy #28 in Table 2). Panelists also did not agree on the importance of three items: (1) *Develop/research existing point of care resources for survivorship care* (#22); (2) *Implement a project ECHO* (*Extension for Community Healthcare Outcomes*) *on breast cancer survivorship for primary care clinicians* (#24; project ECHO consists of a virtual learning community of primary care physicians, specialists, and staff who would join a series of 60‐min Zoom sessions discussing real‐life patient scenarios); and (3) *Implement patient support groups/group visits for breast cancer survivors in primary care* (#29).

### 3.2 Most important, uncertain feasibility

Panelists agreed that three priority items were most important. These were the only items to be scored a 9 on the 1–9 scale following Round 3:

- *Define clear roles and responsibilities for both oncology and primary care for patients with a history of breast cancer* (#1).
- *Train oncologists about how primary care can address survivorship care* (#2).
- *Create clear pathways for communicating and sharing information with oncology* (#3).

Notably, all three items prioritize the theme of “oncology‐primary care connections,” highlighting the need to clarify the roles and responsibilities of primary care and oncology and strengthen their communication and relationships. While panelists indicated improving primary care‐oncology connections was especially important, they were uncertain about the feasibility of these items (scored 5 or 6).

### 3.3 Important and feasible strategies

Panelists agreed that four priority items were both important and feasible (scored 7 or 8 on both criteria):

- *Educate patients on survivorship* (#4).
- *Enable screening reminders, monitoring alerts, and automated messaging within electronic health records* (*EHRs*) (#5).
- *Identify available resources for primary care clinicians to recommend to patients/families* (#6).
- *Develop and/or distribute accessible clinician reference guides detailing common breast cancer drugs and effects* (#11).

These items highlight three of the five identified thematic groupings: “patient education,” “technological solutions,” and “resource development and distribution.”

### 3.4 Importance

Overall, panelists who participated in both rounds evaluated the importance of most items favorably, with only one item – *Implement patient support groups/group visits for breast cancer survivors in primary care* (#29) – scored as not important, although panelists did not reach agreement on this (as noted above). For items where consensus was eventually reached, panelists evaluated 18 items as important and eight items as uncertain. After the final evaluation round, all five items to build connections between oncology and primary care (#s 1, 2, 3, 9, and 17) were evaluated as important.

Four of the seven “primary care practice trainings” (#s 10, 13, 14, and 16) were assessed as important and emphasized knowledge of breast cancer treatment and clinical guidelines (i.e., treatment effects, surveillance, screenings). Two of the five “technological solutions”—creating automated EHR reminders (#5) and standardizing cancer history information (#7)—were identified as important. Two of the four “patient education” items, aiming to improve patients' survivorship knowledge (#4) and their skills communicating with primary care clinicians (#8), were evaluated as important. Four of the six “resource development/distribution” items were deemed important and included resource lists for patients and families (#6), the development of clinician reference guides (#11) and risk‐stratification guidelines (#15), and the inclusion of patient‐reported outcomes on intake forms (#12). Only one of the four “system‐level strategies” to provide coordinators to facilitate survivorship care (#18) was identified as important.

Round 3 only panelists (*n* = 5) generally agreed with the larger panel's assessments of importance with a few exceptions. Three items assessed as important by the larger panel were evaluated by Round 3 only panelists as uncertain:

- *Identify available resources to recommend patients/families* (#6).
- *Work with oncology to develop primary care‐friendly plans* (#9).
- *Work with oncology to engage primary during acute treatment* (#17).

Two items, which the larger panel agreed were uncertain, were evaluated as important by Round 3 only panelists: *Train primary care clinicians on how to use point‐of‐care resources to find information* (#26) and *develop a patient registry for cancer survivors in the EHR* (#27).

### 3.5 Feasibility

The larger panel agreed that the feasibility of most (21/29) items was uncertain. Panelists assessed only five items as feasible after Round 3. Four of these items were also evaluated as important (discussed above). One additional item was evaluated as feasible and of uncertain importance: *Design primary care clinician‐friendly templates for follow‐up plans in EHR* (#19).

Panelists also agreed that two priority items were not feasible: *Provide managers/coordinators to facilitate survivorship care* (#18) and *Implement patient support groups/group visits for breast cancer survivors* (#29). Panelists did not positively evaluate the feasibility of any items that were thematically categorized as “primary care practice trainings” or “system‐level solutions.”

Round 3 only panelists shared similar views to the larger panel regarding feasibility with four exceptions. Round 3 only panelists evaluated all four of the exceptions as more feasible than the larger panel:

- *Educate patients on survivorship care strategies and how to communicate care needs with primary care clinicians* (#8).
- *Add patient reported outcomes to intake forms* (#12).
- *Provide managers/coordinators to facilitate survivorship care* (#18).
- *Document consistently where history of cancer is located in EHR* (#21).

## 4 DISCUSSION

Overall, panelists reached consensus about the importance and feasibility of most priority items after Round 3 with a few exceptions. Panelists identified 18 of the 29 items as important, indicating the value of varied priority areas with which to consider tailoring to context and implementation. However, they were less optimistic about feasibility: following Round 3, they assessed 21 of the 29 items as uncertain. This may have stemmed from the fact that prompts were framed for panelists to elicit priority areas broadly rather than in a specific context. However, it may also reflect the inertia of translating primary care‐engaged survivorship models into practice in the U.S. despite advancements of implementing these models in other countries.^35^ Previous critiques of strategies that have failed have re‐directed survivorship experts to pay closer attention to context and processes rather than favoring static approaches (e.g., guideline development, survivorship care plans).^11^, ^36^ This was the impetus for this study—to identify priority areas that can be tailored to context that support breast cancer survivorship care in primary care settings. Our findings regarding the widespread uncertainty about the feasibility of most priority items (including those most important) indicate that stakeholder engagement and capacity building may be needed to render important priority items actionable.

To that end, our findings reveal two important takeaways. First, primary care clinicians and oncologists have been found to hold divergent views when it comes to communication and their respective responsibilities for survivorship care,^37^ illustrating the gulf that currently exists. This is not a problem unique to cancer, as primary care generalists and specialty care physicians tend to operate from different clinical orientations.^38^ To date, efforts to address the cancer survivorship care gap with primary care are characterized as “onco‐centric,” favoring a specialty care orientation that aims to: (1) identify disease for management; (2) interpret what needs to happen through specialized knowledge; and (3) develop and carry out a care plan.^38^ In contrast, the generalist orientation (1) recognizes problems and assesses capacity; (2) prioritizes attention and action to foster health and connection; and (3) personalizes care based on patient priorities.^38^ Panelists recommended a set of priorities that would shift both generalist and specialist perspectives by aiming to clarify roles for both groups, improve communication, and foster productive relationships between these groups. Among the 31 priority items evaluated, panelists agreed that these items were the most important (scoring 9 on importance). In contrast to many of the recommended priority areas, these items require cross‐team collaboration and unbillable time, and thus, unsurprisingly, panelists were uncertain about their feasibility. These priority areas call for cross‐discipline capacity building and shared understandings, which have been absent from previous cancer survivorship implementation efforts. Notably, these items ranked as most important do not emphasize static approaches, such as developing additional tools or disseminating guidelines. Additionally, they emphasize the need for knowledge translation that honors both specialist and generalist orientations rather than emphasize deficits in primary care.

Regarding the second important takeaway, four priority areas were evaluated as both important and feasible. Delphi items that are positively assessed along all criteria (in this case as both important and feasible) are often deemed “preferred.”^39^ However, when considering how to effectively improve survivorship care, we approach these items with caution. These items recommend offloading responsibility to patients, who are already caught in the middle, having to coordinate the exchange of information between oncology and primary care; automating information and tasks; and providing ready‐made resources that clinicians can access as reference tools that require less extensive investment than other educational recommendations (e.g., training clinicians on survivorship care guidelines). Although empowering patients and promoting patients' self‐management are important goals, by intentionally shifting the responsibility to coordinate care in a fragmented delivery system to patients, these priority items may exacerbate disparities in cancer survivorship care, especially for patients with greater care complexity (due to social need or chronic conditions).^40^ Although these priorities may require relatively limited investment and collaboration, rendering them more “actionable,” they may achieve limited long‐term success given that they will simply saturate survivorship care with yet more tools that have not been well received.

Although panelists questioned the feasibility of how primary care‐oncology connections might be fostered, investing in these priorities may have lasting impact. As experts who are directly involved in developing survivorship programs and delivering care to survivors, these panelists are well‐positioned to consider the processes and structures needed to help tailor and implement tools and guidelines that have yet to be effectively adopted. Having identified panelists' priorities, consideration of how to tailor and implement them is a crucial next step. Additionally, mapping these priority areas onto the Expert Recommendations for Implementing Change (ERIC) framework will help operationalize them in practice.^41^ Effectively implementing these changes to improve shared understandings across primary care and oncology may be the foundation of many needed changes. This capacity building could have a ripple effect that would make the tailoring and implementation of other important priority areas more feasible. For example, with better communication and understanding of roles, standardizing cancer history information in the EHR and engaging primary care during acute treatment can be more easily managed. Perhaps most importantly, facilitating stronger relationships between primary care and oncology can encourage paradigm shifts for both clinician groups, further supporting the coordination and collaboration between primary care and oncology that is currently lacking but necessary for long‐term sustainable change.

This study is not without limitations. Sampling experts currently working in and developing primary care‐engaged survivorship programs provides perspectives that may not resonate with on‐the‐ground primary care clinicians. As such, we do not know if their views generalize to primary care more broadly. Nevertheless, given their deep knowledge of cancer care and ongoing efforts to bridge to oncology, their expertise brings valuable understandings about what is required to effectively fill gaps primary care is currently facing when providing care to patients with a history of breast cancer. The sample size of the study was also quite small, limiting our understanding of the representativeness of these experts' perspectives. However, given that this specialized population is not large to begin with and that they reached consensus on many issues provide meaningful insights. The sample size is also consistent with other Delphi studies.^24^, ^32^ Additionally, although we asked panelists to consider breast cancer survivorship care, what we learned from them spans the survivorship continuum and is likely relevant to other cancer care contexts.

## AUTHOR CONTRIBUTIONS

**Lisa Mikesell:** Formal analysis (lead); investigation (equal); methodology (lead); supervision (equal); writing – original draft (lead); writing – review and editing (lead). **Denalee M. O'Malley:** Formal analysis (supporting); writing – original draft (equal); writing – review and editing (equal). **Rachel T. Kurtzman:** Project administration (equal); resources (equal); writing – review and editing (supporting). **Jenna Howard:** Formal analysis (supporting); writing – original draft (supporting); writing – review and editing (equal). **Benjamin Bates:** Formal analysis (supporting); writing – original draft (supporting); writing – review and editing (equal). **Jennifer R. Hemler:** Formal analysis (supporting); writing – original draft (supporting); writing – review and editing (equal). **Sarah J. Fadem:** Formal analysis (supporting); writing – original draft (supporting); writing – review and editing (equal). **Jeanne M. Ferrante:** Formal analysis (supporting); writing – original draft (supporting); writing – review and editing (equal). **Alicja Bator:** Project administration (lead); writing – review and editing (supporting). **Shawna V. Hudson:** Conceptualization (lead); formal analysis (supporting); funding acquisition (lead); methodology (supporting); supervision (equal); writing – review and editing (equal). **Benjamin F. Crabtree:** Conceptualization (lead); formal analysis (supporting); funding acquisition (lead); methodology (supporting); supervision (equal); writing – review and editing (equal).

## CONFLICT OF INTEREST STATEMENT

The authors have no conflicts of interest to report.

## ETHICS STATEMENT

The Rutgers University IRB approved the study protocol (Pro 2021000838) and electronic consent was obtained from all participants.

## PRECISE

Based on Delphi panel findings, U.S. experts working in primary care engaged survivorship programs agreed that supporting survivorship care requires improved communication and connections between primary care and oncology. Although primary care‐oncology relationships were prioritized as most important among 31 recommended priority items evaluated, panelists were uncertain about their feasibility.

## Data Availability

The data that support the findings of this study are available from the corresponding author upon reasonable request.
